# Resilience of health-care workers in the UK; a cross-sectional survey

**DOI:** 10.1186/s12995-015-0061-x

**Published:** 2015-05-21

**Authors:** Andeep Sull, Nicholas Harland, Andrew Moore

**Affiliations:** NIHR Newcastle Biomedical Centre & Unit, Newcastle University, Campus for Ageing & Vitality, Newcastle, UK; MSK Services, Friarage hospital, Northallerton, UK; Corporate Improvement Team, James Cook Hospital, South Tees Hospitals NHS Foundation Trust, Middlesbrough, UK

## Abstract

**Background:**

Working for the UK National Health Service (NHS) requires working for organisations under financial pressures and frequent restructures, which can lead to anxiety over continuing employment and income. There are currently no studies to date that have examined the influence of personal resilience across all professions and demographics in the NHS. This study aims to quantify resilience within an NHS trust and explore the contribution of demographic variables of gender, age, years of service, pay grade, hours worked, job role, and division worked to the resilience response of employees. The study also explores the relationship between resilience levels and absence rates, as a marker for health and well-being amongst NHS staff.

**Methods:**

This study consists of a cross-sectional on-line survey of staff employed in an NHS Trust. All trust employees were asked to complete a Resilience Scale (RS-25), and demographic questions including age, sex, length of service, NHS pay grade (banding), division, job role and number of hours worked per week. Trust level sickness absence rates were also collected during this period. Results were analysed using descriptive statistics, bivariate comparisons and chi-squared tests.

**Results:**

Data was gained from 845 employees; a significant association between gender and resilience found females scoring higher on the resilience scale; *x*^2^(5) =18.30, p < 0.05. A weak positive correlation between age and resilience found older employees displaying a higher level of resilience; r = 0.11, p <0.05. Results also suggest employees working between 18.75-37.5 hours a week have higher levels of resilience. Ancillary staff scored low on resilience compared to all other staff groups which showed moderate resilience. Clinical staff scored lower on resilience compared to both administrative staff and clinical staff with line management responsibilities. No correlations were found between absence rates and resilience.

**Conclusion:**

This study gives a snapshot of the resilience of employees in a NHS trust. It is the first of its kind to take into consideration all job roles, divisions and the banding system within a trust. The results also indicate that resilience levels may not be a mediating factor for the health and well-being of NHS staff.

## Background

Working in an NHS healthcare environment is becoming increasingly tough due to the financial pressures, organisational restructures, ageing population, long working hours and anxiety over continuing employment and income. This can result in increased feelings of complexity and ambiguity for staff [1]. Given this context keeping ourselves healthy and being able to adapt to change is critical to both personal wellbeing, and the ability to support organisations provide high quality services.

The National Health Service (NHS) is one of the largest employers in the world, and is the biggest in Europe, with over 1.3 million staff, with net expenditure increasing from £57.049 billion in 2002/03 to £105.254bn in 2012/13 [2]. The number of doctors employed by the NHS has increased by an annual average of 3.0 per cent since 2003 [2]. From a research perspective therefore the potential impact of large sample baseline data as a means of informing future research questions and studies is significant.

Stress related work loss is estimated to cost the UK economy more than £15 billion a year in revenue and 140 million working days lost [3]. Personal or work stress can lead to a psychological syndrome labelled as ‘burn out’ which manifests as exhaustion, cynicism, and feeling little personal accomplishment [4]. Burn out can lead to feelings of reduced commitment and increased disengagement with work, which in turn can lead to short or even long term sickness absence [3-5]. Some individuals are more ‘resilient’ than others, and therefore likely to cope better with challenging times, and therefore remain in the workplace [1].

There are many definitions of resilience; it is generally described as the ability of an individual to successfully adapt, maintain competent functioning, and ‘bounce-back’ from adversity and major life stressors [1,6,7]. Emotional resilience is defined as an individual’s ability to adapt to various adverse conditions while maintaining a sense of purpose, balance and positive mental and physical wellbeing [8]. Resilience is the capacity to not only survive life's challenges, but to learn and grow from them, and become stronger as a result of such challenges. This capacity, a key factor in determining how people will respond to change, is something that can develop over time in the right circumstances [1].

Resilience research has historically focused upon individual, child, family, or community resilience [9]. More recently however research has begun to explore the employee and organisational impact of resilience, pointing to the role of resilience as a positive organisational factor which can be developed and which yields significant individual and organisational benefits including improved productivity, improved wellbeing, and reduced absenteeism and turnover [3]. Literature suggests that resilient individuals are better equipped to deal with the stressors that result from a constantly changing work environment. They are more likely to make sound decisions in critical moments, and less likely to be off sick or chose to leave the organisation due to organisational stressors saving the organisation money [6].

The case for organisations to support personal resilience learning interventions within the workplace is strengthened based on the importance of staff well-being and its’ impact on patient care being well-evidenced in recent research. A recent report indicates a need for the work stating that wellbeing initiatives need to involve the enhancement of positive psychological wellbeing (for staff) as well as the reduction of negative pressure i.e. stress-coping strategies and social support are important factors to incorporate into such initiatives [10].

The NHS Health and Wellbeing Review [11] describes the importance for all UK NHS trusts to develop a clear strategy and vision for the future to improve staff health and wellbeing. The report also identified clear links between staff health and wellbeing and patient safety, patient experience and the effectiveness of patient care. Whilst it is difficult to keep staff health and wellbeing at its optimum at a time when there are cutbacks, constant changes and restructures within the workplace, it is at these times the focus is imperative. Research suggests however that nurses in particular can reduce their vulnerability to workplace adversity by the development and strengthening of their own personal resilience [12]. These authors also recommend that resilience training is incorporated into nursing education and that professional support should be encouraged through mentorship programmes. Sergent & Laws-Chapman also agree that resilience training should be enforced amongst NHS trusts to help create healthier workplace cultures, reduce absenteeism due to poor health and well-being, improve teamwork and raise morale [8].

There are currently no studies that have examined the influence of work based demographics on resilience across all professions in an NHS healthcare trust. Resilience has been studied within specific occupational groups in healthcare such as psychiatric nurses, mental health clinicians, paediatric and neonatal intensive care unit medical staff, and operating room nurses [13-16]. However, none of these studies have explored the role of work based demographic variables in resilience across a large sample within a NHS trust. This study adds to the current literature regarding resilience by examining different groups within an NHS workforce and their resilience characteristics.

The aims of this study were three fold; to quantify resilience within a UK NHS trust, to explore the contribution of work based demographic variables to the resilience of employees of a NHS trust and to explore the relationship between resilience levels and absence rates as a marker for health amongst healthcare workers. Overall the motivating factor for conducting this research is to create a large resilience dataset that can be used by practitioners and researchers in ‘Organisational Development’ teams within healthcare trusts to aid their decision making process when introducing health and wellbeing interventions for their healthcare staff. Data from the survey could strengthen the case for Personal Resilience training within NHS organisations in line with the Department of Health (DH) initiative “Healthy staff, better care for patients” [17].

## Methods

### Study design and setting

This study consisted of a cross-sectional on-line survey of staff employed in an NHS Trust based in Northern England. The study was approved by the participating NHS Trusts Research Development and Governance unit as a service evaluation rather than research; therefore no ethical approval was needed. The resilience questionnaire was distributed through an online survey platform known as Survey Monkey. Although no individual consent forms were needed, participants were informed before commencing the survey that no identifiable data would be collected and the data that was collected would be kept confidential and password protected.

### Sample and data collection

All staff employed by the trust were eligible to be sent the survey questionnaire, there were no exclusion criteria. A top down email cascade approach was used to send the survey link through established trust information dissemination pathways. To ensure a high level of saturation the cascade approach was repeated 4 times, at the start of each consecutive month from August 2013- November 2013. The link was also issued in the trust’s monthly newsletter during the same 4 consecutive months.

#### Measures

Employees were sent a survey link consisting of;

1. 25-item Resilience Scale (RS-25) [18]
  This measures an individual’s resilience score, which can range from 25 (very low resilience) to 175 (very high resilience). Respondents are asked to rate how they feel on a 1-7 point scale anchored with “strongly disagree” to “strongly agree” in answer to a question such as “I feel I can handle many things at a time” and “I usually look at a situation in a number of ways”. The questionnaire has five sub-scales that can be scored separately; a purposeful life, perseverance, equanimity, self-reliance and existential aloneness.
  The Overall RS25 score is categorised into 6 levels; very low (25-100), low (101-115), moderately low (116-130), moderate (131-144), moderately high (145-160) and very high (161-175).
  Reliability was not measured in the current study, as the Resilience Scale has been widely used and been shown through psychometric testing to be a valid and reliable measure [19]. The internal consistency reliability for the Resilience Scale was found to be .89, and the coefficient alpha .91 which is considered satisfactory [20].
2. Demographic questions included employee’s age, sex, length of service, NHS pay grade (banding), division, job role and number of hours worked per week. Job role was determined by using 6 items: administrative, admin with line management, ancillary, clinical, clinical with line management and management. In the UK, a ‘banding’ system exists that relates to every non-medical job within the NHS, e.g. excludes doctors and consultants that have a separate pay structure [21]. This banding ranges from band 1, the lowest paid and least qualified worker, to band 8c the highest qualified and paid worker.
3. Absence figures were collected for the trust during these months, and these were split according to age, sex, length of service, band and division.

### Analysis

Firstly, descriptive statistics were performed to provide background information on the sample. Bivariate comparisons were then carried out between different items and their resilience scores using t-tests. Chi-squared test were used to compare any categorical data E.g. division and job role. Finally analyses examined associations between resilience scores and absence rates for each of the variables.

## Results

### Sample

Data was gained from 845 employees, 789 of which completed all the data. Males compromised 21% of the respondents and 79% female, which is representative of the national NHS workforce (female 77%, male 23%)[22]. 66% of respondents were aged between 40-59, 57% had worked in the service between 11-30 years, and 73% of the sample worked full time. For this sample the mean score on the RS-25 was 135.5 (sd = 19.7). RS-25 (2010) on-line sample from the general population reported the same mean score of 135.5 (sd = 19.7) [18].

### Response rate

The response rate of this survey is unknown due to the cascade approach which was used to collect data. From the 845 employees that completed the survey we can see that response rates gradually declined over the 4 months as expected (August: 44%, September: 25%, October: 20%, November: 11%).

### Resilience scores

#### Gender

Table 1 shows males scored an average of 130.8 (sd = 24.5), rounded up this demonstrates a moderate level of resilience. Females scored slightly higher with an average of 136.6 (sd = 18.1), also indicating a moderate level of resilience but both male and female scores being at the very bottom of the moderate resilience range. Pearson’s chi-square tests shows a significant association between gender and rating of resilience score *x*^2^(5) = 18.30, p < 0.05 (Table 2).

**Table 1:** **Resilience scale results for subgroups**

**Sex**	**Resilience score** **(mean)**	**Standard deviation**
Male	130.77	24.538
Female	136.64	18.121
**Age**		
18-24	117.33	42.141
25-30	132.55	16.237
31-39	133.75	20.315
40-49	136.10	17.718
50-59	137.52	19.299
60+	137.13	27.133
**Length of years service**		
Less than a year	138.18	20.255
1-2 years	135.57	16.566
3-5 years	139.12	24.357
6-10 years	130.77	18.611
11-20 years	136.09	19.545
21-30 years	135.26	18.564
31-40 years	137.84	16.854
40+ years	133.07	34.012
**Hours**		
Full time	135.08	20.004
Part time (18.75 or less)	124.03	25.491
Part time (18.75 or above)	138.90	16.622
**Band**		
1	128.80	24.479
2	137.42	18.591
3	133.97	23.322
4	139.08	19.556
5	133.43	23.748
6	133.74	18.491
7	136.61	18.061
8	137.87	16.275
Medical	130.44	24.042
**Job Role**		
Administrative with Line Management	136.96	17.075
Ancillary	122.50	27.948
Clinical	133.31	18.655
Clinical with Line Management	137.88	16.184
Management	135.83	20.215
**Division**		
Academic	140.34	18.329
Acute Medicine	137.53	18.612
Anaesthesia and Theatres	138.45	15.253
Cardiothoracic Services	137.19	17.634
Chief Executives Office	150.11	10.856
Clinical Support Services	130.68	21.696
Community Services	135.11	16.126
Finance Directorate	141.14	21.348
Human Resources	135.15	25.841
IT and Health Records	140.86	17.060
Neurosciences	137.56	20.402
Operational Services	131.94	27.461
Pathology	129.10	19.839
Planning	125.38	11.338
Quality Assurance	138.03	11.491
Radiology	133.75	14.064
Speciality Medicine	132.29	18.346
Surgery	136.78	22.298
Trauma	144.23	16.729
Women and Children	130.68	25.790

**Table 2:** **Pearson**’**s**-**chi square test for association between gender and resilience rating**

	**Value**	**df**	**Asymp.Sig.** **(2-sided)**
Pearson Chi-square	18.299*	5	.003
Likelihood Ratio	16.438	5	.006
Linear-by-Linear	8.600	1	.003
No of Valid Cases	784		

*0 cells (.0%) have expected count less than 5. The minimum expected count is 5.88.

#### Age

Table 1 clearly shows the respondents resilience score increases with age. Upon correlational analysis a weak positive correlation between the age and resilience was found, r = 0.11, p <0.05 (Table 3).

**Table 3:** **Spearman**’**s Correlation for relationship between age and resilience score**

		**Resilience score**	**Age**
Resilience score	Correlation Coefficient	1.000	.113**
	Sig. (2-tailed)	.	.002
	N	789	786
Age	Correlation Coefficient	.113**	1.000
	Sig. (2-tailed)	.002	.
	N	786	786

**Correlation is significant at the 0.01 level (2-tailed).

#### Length of service

Table 1 shows that resilience scores fall within the moderate range regardless of length of service. Correlational analysis indicates no relationships between length of service and resilience.

#### Hours worked

Table 1 shows staff employed to work below 18.75 hours have a low resilience score, whilst employees working full time (37.5 hours) or between 18.75 hours and part time have a moderate resilience score. Independent samples t-tests were carried out and found significant relationships between all three factors (Table 4). On average, part time staff working less than 18.75 hours have a statistically significantly lower level of resilience than full time staff t(606) = 0.26, p < 0.05 and staff working more than 18.75 hours t (213) = 4.26, p < 0.05. Part time staff, working above 18.75 hours, scored on average a higher level of resilience than full time staff t (755) = -2.33, p < 0.05.

**Table 4:** **Independent t**-**tests for number of hours worked**

	**Full time**	**Above 18.75 hours**	**Below 18.75 hours**
Full time		p = 0.20 T = -2.327	p = .003 T = .259
Above 18.75 hours			p = .000 T = 4.259
Below 18.75 hours			

#### Banding

Band 1 and medical staff both showed low resilience. All other bands (2-8) scored had moderate resilience with scores ranging from 133–139 (Table 1). Further analysis indicates no significant relationship between an employee’s band and resilience level.

#### Job role

Table 1 shows Ancillary staff scored low on the resilience compared to all other staff groups of staff which all show moderate resilience. Independent t-tests were carried out to compare the mean scores of each of the items within job role and the results of this analysis can be seen in Table 5. On average Ancillary staff scored lower on resilience than all other staff groups. Clinical staff scored lower on resilience compared to administrative staff and clinical with line management staff. There were no other statistical differences found between any other staff groups.

**Table 5:** **Independent t tests for job role and resilience score**

	**Administrative**	**Admin with line management**	**Ancillary**	**Clinical**	**Clinical with line management**	**Management**
Administrative		t = .539 sig = .591	t = -3.603 sig = .000**	t = 2.797 sig = .005**	t = .405 sig = .686	t = 1.138 sig = .256
Admin with line management			t = -2.895 sig = .005**	t = 1.302 sig = .194	t = -.344 sig = .731	t = .347 sig = .729
Ancillary				t = -2.069 sig = .047	t = -4.120 sig = .006**	t = -2.956 sig = .004**
Clinical					t = 2.519 sig = .012*	t = 1.192 sig = .234
Clinical with Line Management						t = .910 sig = .363
Management						

*significant to p < 0.05 **significant to p < 0.01.

#### Division

Table 1 shows the only division that scored moderately high was the chief executive’s office with resilience mean score of 150 (sd = 10.9), and the only divisions scoring low were pathology with an average of 129 (sd = 19.9) and planning with a mean resilience score of 125 (sd = 11.3). All other divisions scored in the moderate range. Chi squared could not be used to analyse further as over 20% of variables did not have the necessary count of 5 or more.

### Absence rates

Correlational analysis was used to see if there was a relationship between resilience scores and absence rates in the following groups; gender, age, banding, length of service and division. No correlations were found between any of the variables and resilience.

## Discussion

This study sought to collect a large sample cross-sectional data regarding resilience within a NHS trust. The resilience scores of employees of the trust ranged from 25 to 175, and the average resilience level of the trust was moderate. A moderate level indicates neither high nor low resilience, and although individuals at this level might possess many characteristics of resilience, these need building upon and strengthening [18].

The data in this sample showed that female employees scored higher on resilience than males. This adds to the current literature of resilience in a workplace as no studies could be found with similar findings [18]. The sample also showed a small correlation between age and resilience, which is consistent with findings [16,18,23] that suggest staff develop resilience through a life span though. Findings in this area vary as there is also research to suggest effects of increased age are not associated with higher levels of resilience [24].

In a sample of Australian Operating Room nurses, it was found five variables (hope, self-efficacy, coping, control and competence) explained resilience at statistically significant levels whilst age, experience, education and years of employment did not contribute to resilience [25]. A similar survey was carried out again in 2009 [16], however this time a statistical significant relationship was found between age and resilience, and years of experience and resilience. Although the association was small, it is similar to the results from the current survey. The inconsistencies of the relationship between age and resilience need to be studied further possibly using longitudinal studies where employees can be tested at several points in their career.

Findings from analysing the job roles of employees suggest that ancillary staff experienced low resilience, lower than all other job roles, and clinical staff reported lower levels of resilience than administrative and clinical staff with line management responsibilities. The fact that both clinical and ancillary staff reported lower levels of resilience than administrative relates to a study that showed that both these staff groups also scored higher on a perceived pressure scale than administrative staff [26]. The relationship between perceived pressure and resilience level within specific job roles should also be looked at further in future studies. One possible explanation for ancillary and clinical staff scoring lower on resilience is because their coping strategies suggest they make little use of within work social support compared to administrative staff [26]. Whether this may be related to available social time and/or roles involving constant movement between different hospital areas remain uncertain. Findings by Edward [2005] also suggest that a supportive team is related to resilience [14]. A recent study also supports the idea that a supportive superior as well as relationships with colleagues seem to be an important factor for physical wellbeing at different career levels [27].

Findings also suggest employees working part time below 18.75 hours experience low resilience compare to employees working over 18.75 hours. Yet employees working above 18.75 hours but not full time (37.5 hrs) experience higher resilience than those working full time. It could be inferred that individuals working less hours might have a lower income and also lower level of education, however findings from previous research suggests there is no significant relationships between income, education level and resilience [16,23]. With a very dominantly female population however it could be hypothesised that some staff reduce hours to a degree to improve their work-life balance reducing their stress and therefore increasing their resilience. No research could be found to support these findings or hypothesis however and therefore this article suggests that future studies should look at factors that affect chosen work hours and whether these factors positively or negatively impact resilience and health.

No relationships were found between absence rates and any of the examined variables. This is counter to the findings of Rees 1995 who showed, in a smaller sample, that clinical staff were less likely to take time off sick as a result of stress than non-clinical staff [26]. Although individuals might not take time off work, stress can affect their focus, attention to detail and behaviour [28]. A recent paper produced for Ashridge Business School suggests further work would be worthwhile to assess the impact of resilience training interventions on absenteeism through longitudinal study [29].

Recent research suggests resilience building strategies such as seeking mentoring relationships, achieving life balance and spirituality, positive emotions and personal growth and reflection were found to have protective factors that can help individuals achieve positive personal outcomes [12]. This study suggested the organisational benefits of building personal resilience include lowering vulnerability to adversity, improved well-being and achieving better care outcomes and lowering the trend of nurses leaving the healthcare system. A firm recommendation from study is for resilience-building to be incorporated into nursing education and that professional support should be encouraged. Results from the current study would agree with this, however suggest that professional support and mentorship should be encouraged across all staff groups, especially clinical and ancillary staff who are often working in isolation.

Another resilience building strategy which has been recently studied is ‘Building Personal and Professional Resources of Resilience and Agility in the Healthcare Workplace’. Positive coping strategies were taught in a workplace intervention and seen as feasible and effective in producing statistically significant outcomes. The intervention ran for seven months and was specifically aimed at oncology staff and healthcare leaders [30]. Recent research in the personal resilience of nurses and midwives suggest work-based, educational interventions that focus on personal resilience have significant potential to empower both clinicians and students to withstand the workplace adversity they will no doubt face at some point in their career [31], especially in the current climate [1].

There are currently no studies to date that have examined the introduction of a programme of employee personal resilience interventions across different staff groups within an organisation. Studies to date demonstrate the introduction of one specific learning intervention within a specific staff group. The results from the current study suggest that different staff groups have significantly different levels of resilience, therefore moving forward resilience work should focus on developing a programme of resilience interventions through longitudinal study which are specific to different staff groups within a healthcare setting.

Interpretation of our study findings is subject to certain limitations. The sample was drawn from only one NHS trust, therefore the findings may not be representative of trusts across the UK, as different organisations have different cultures which could impact on resilience ratings. There may also have been other demographic variables that were not measured in this study or variables that could be categorised differently that could have had an impact on resilience levels such as education level, marital status and number of previous jobs held. Health and well-being were only examined as related to work absence in this large sample study but future studies would be advised to use more specific measures, though achieving this in a large sample can be challenging. A longitudinal study on resilience would also help to study the relationship between the demographic variables of employees and their resilience over time.

## Conclusions

This study gives a snapshot of the resilience of employees in a NHS trust. It is the first of its kind to take into consideration all job roles and divisions within a trust, it suggests that resilience alone cannot be measured as a marker for health as related to sickness absence amongst NHS staff.
